# The coexistence of pulmonary tuberculosis and adult-onset Henoch–Schönlein purpura

**DOI:** 10.1093/rap/rkaa039

**Published:** 2020-08-08

**Authors:** Adnan N Kiani, Sharon E Nunez, Frank O’Sullivan, M Muruganandum, Nicole S Emil

**Affiliations:** Division of Rheumatology, Department of Medicine, University of New Mexico, Albuquerque, NM, USA

**Key message**

- *Mycobacterium tuberculosis*, albeit rare, can trigger Henoch-Schönlein purpura.

Sir, Henoch–Schönlein purpura commonly presents in children and adolescents between 3 and 15 years of age and is more frequent among boys than girls (male-to-female ratio 1.5:1) [1]. It is an IgA-mediated immune vasculitis that varies in severity and target organ involvement. The mildest forms can be limited to the skin, whereas more severe cases involve the kidney and gut. Diagnosis is usually clinical, based on abdominal pain, haematuria, arthritis and non-thrombocytopenic purpura. A purpuric skin rash (palpable purpura), usually on the lower extremities, is generally necessary for diagnosis. Henoch–Schönlein purpura is relatively uncommon but tends to be more severe when it occurs in adults. Several precipitating factors have been implicated in the development of Henoch–Schönlein purpura, including malignancy, infections, environmental chemicals, toxins, physical trauma and complement deficiency [2, 3]. In a recent report of 420 paediatric Henoch–Schönlein purpura patients, arthralgias and renal involvement were common, with colchicine being an effective treatment in relapsing patients [4]. Here, we report an unusual case of Henoch–Schönlein purpura associated with pulmonary tuberculosis in a young adult with no pulmonary symptoms.

**Fig. 1:**
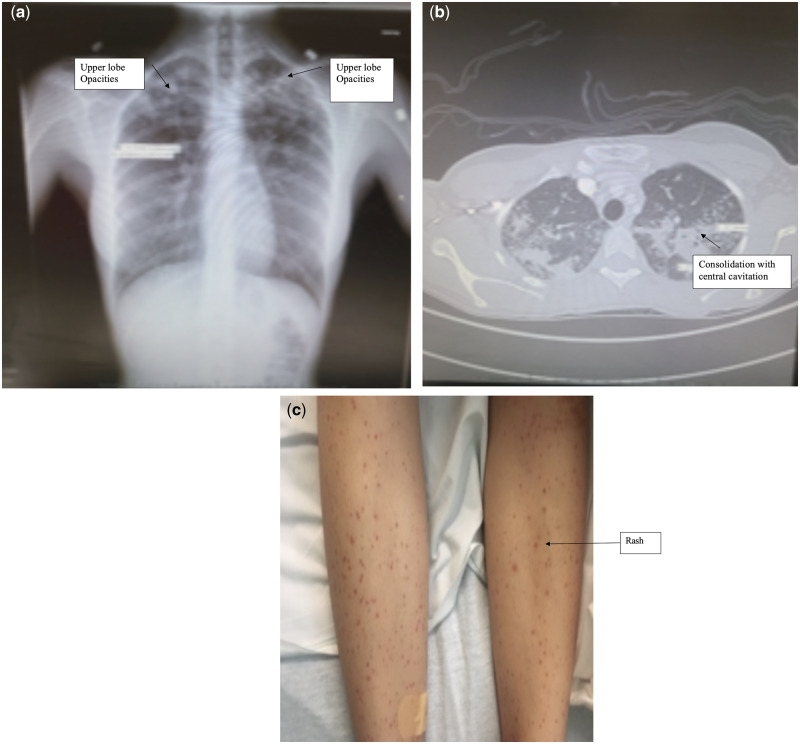
Chest XRAY showing bilateral upper lobe opacities

A 21-year-old woman with no significant past medical history presented to our hospital with a 1 month history of bilateral lower extremity petechial rash (Fig. 1C). The rash first appeared after working a shift at a fast food restaurant. At that time, it was asymptomatic and resolved within 3 days. Over the next few days, the rash recurred and eventually developed into an extensive, painful eruption extending from the mid-thigh to the ankles. She had not experienced recent infection, fever, chills, weight loss, pleuritic symptoms, photosensitivity, alopecia or mouth, nasal or genital sores. She denied any shortness of breath, epistaxis, haemoptysis, haematemesis, haematochezia or frequent sinus infections. There was no history of contact with individuals with contagious diseases. In the emergency department, laboratory evaluation revealed an ESR of 80 mm/hr (normal range, 0–28) and CRP at 4.5 (normal, <0.3). Further testing, including ANCA, C3, C4, complete blood count, comprehensive metabolic panel (CMP), urinalysis (UA), urinary protein and creatinine, were negative or within normal limits.

A skin biopsy revealed IgA vasculitis consistent with Henoch–Schönlein purpura. Chest X-ray was abnormal, with bilateral upper lobe opacities (Fig. 1A). Chest CT revealed upper lobe predominant patchy and confluent consolidation, with central cavitation in the left upper lobe (Fig. 1B). Further testing revealed a positive IFNγ release assay for tuberculosis, and sputum smears were negative for acid-fast bacilli. However, PCR testing of blood for *Mycobacterium tuberculosis* was positive ×2 with PCR twice. The patient was discharged on combination therapy with rifampin, isoniazid, pyrazinamide and ethambutol, including 2 months of intensive-phase treatment. Subsequently, she experienced resolution of the rash, and sputum smears have remained negative for acid-fast bacilli. Continuation-phase treatment with rifampin and isoniazid for an additional 4 months was prescribed, along with local health department monitoring.

The aetiology and pathogenesis of Henoch–Schönlein purpura are not completely understood. It is presumed that exposure to various antigens, such as infectious agents, dietary allergens, toxins and drugs, are immunological triggers. Several abnormalities relating to IgA have also been implicated, including IgA immune complexes, IgA RF, elevated serum IgA concentrations, and deposition of IgA in affected organs. Complement abnormalities, including depressed CH50 and properdin levels and elevated C3, C3d or decreased C3 levels have also been described in the acute phase of the illness [2, 3, 5]. Among the paediatric patient population, renal involvement is common, with a nephrotic/nephritic picture and no consensus on when to perform a renal biopsy. Management includes cytotoxic medications, including AZA, CSs, CYC and MMF [6]. In a large study of 1200 Henoch–Schönlein purpura paediatric Chinese patients, tuberculosis was found to be a triggering factor of Henoch–Schönlein purpura [7]. A similar atypical presentation has been reported in disseminated tuberculosis [8].

In the literature, tuberculosis has rarely been reported in adults with Henoch–Schönlein purpura. In those patients, tuberculosis had a variety of clinical presentations, including pulmonary tuberculosis, pulmonary tuberculosis plus tuberculous lymphadenitis, isolated tuberculous lymphadenitis, tuberculous pleuritis and urinary tract tuberculosis [9–12]. In our patient, Henoch–Schönlein purpura was diagnosed according to clinical findings and biopsy-proven leucocytoclastic vasculitis with IgA deposition. Pulmonary tuberculosis was diagnosed by chest radiograph and CT imaging, sputum smears and PCR testing. Her symptoms resolved with anti-tuberculous treatment without the addition of CSs, suggesting that the Henoch–Schönlein purpura was triggered by her mycobacterial infection.

Here, we report an unusual case of Henoch–Schönlein purpura associated with pulmonary tuberculosis in a young adult with no pulmonary symptoms. Although there is accumulating but rare evidence that active *Mycobacterium tuberculosis* can trigger Henoch–Schönlein purpura, further studies will be required to establish the mechanism whereby this occurs.

*Funding*: No specific funding was received from any funding bodies in the public, commercial or not-for-profit sectors to carry out the work described in this manuscript.

*Disclosure statement*: The authors have declared no conflicts of interest.
